# Hepatoprotection of *Mentha aquatica* L., *Lavandula dentata* L. and *Leonurus cardiaca* L.

**DOI:** 10.3390/antiox8080267

**Published:** 2019-08-02

**Authors:** Olívia R. Pereira, Rocio I. R. Macias, Maria R. M. Domingues, Jose J. G. Marin, Susana M. Cardoso

**Affiliations:** 1 Centro de Investigação de Montanha (CIMO), Instituto Politécnico de Bragança, Campus de Santa Apolónia, 5300-253 Bragança, Portugal; 2 Laboratory of Experimental Hepatology and Drug Targeting, IBSAL, CIBERehd, University of Salamanca, 37007 Salamanca, Spain; 3 Department of Chemistry & QOPNA, University of Aveiro, 3810-193 Aveiro, Portugal; 4 Department of Chemistry & CESAM&ECOMARE, University of Aveiro, 3810-193 Aveiro, Portugal

**Keywords:** *Mentha*, *Lavandula*, *Leonurus*, liver diseases, HepG2 cells, antioxidant, phenolic compounds, UHPLC-DAD-ESI-MS^n^

## Abstract

The phenolic composition of hydroethanolic extracts of *Mentha aquatica* L., *Lavandula dentata* L. and *Leonurus cardiaca* L., obtained from plants grown under organic cultivation, was determined and their hepatoprotective effects were investigated in vitro. *L. cardiaca* extract was rich in phenylethenoid glycosides, especially lavandolifolioside (254 ± 36 μg/mg), whereas rosmarinic acid and eriodictyol-*O*-rutinoside were the major phenolic compounds of *L. dentata* and *M. aquatica* extracts, accounting for 68 ± 7 μg/mg and 145 ± 22 μg/mg, respectively. These differential phenolic components presumably account for their dissimilar antioxidant properties. While *L. cardiaca* extract showed moderate biological effects, *M. aquatica* extract displayed high antioxidant activity in chemical models, and that of *L. dentata* was effective in counteracting potassium dichromate-induced ROS generation in human hepatocarcinoma cells. Moreover, *M. aquatica* extract (50 μg/mL) and its mixture (50%/50%) with *L. dentata* extract displayed an effective cytoprotective effect.

## 1. Introduction

Liver diseases comprise a large number of conditions, either caused by genetic modifications or, more frequently, by viruses, alcohol abuse, toxins or drugs. Liver injury induced by toxins and drugs, haemochromatosis, hepatitis, cirrhosis, and hepatocellular carcinoma are among liver conditions with poor response to available treatments [1].

In recent decades, phytochemicals from plants have been proposed as health supplements acting as preventive agents or for treatment in patients with liver disorders [2]. In this context, polyphenols represent one of the groups with most interest [3] due to their claimed antioxidant and anti-inflammatory properties [4,5]. In fact, oxidative and inflammatory processes are recognized as critical in the pathogenesis and progression of liver conditions [5].

Lamiaceae family is one of the most applied in traditional medicine, as it encloses many plant species which are claimed to exert important pharmacological activities, including antioxidant, antiproliferative, anti-tumoral, anti-inflammatory, antimicrobial, analgesic, and neuroprotective, among others [4]. *Mentha aquatica* L., *Lavandula dentata* L. and *Leonurus cardiaca* L. are three of such species, being used for centuries in traditional medicine for several purposes. In particular, the first is frequently applied in the treatment of external inflammation and in inflammation-related diseases, such as rheumatism, although it is also used as a vermifuge, in the treatment against colds and respiratory problems; to counteract mental illnesses or disorders of the central nervous system; and to attenuate menstruation problems, as a stimulant and as an emetic and astringent agent [6,7,8]. In turn, French lavender, or *Lavandula dentata*, has been used as an antidiabetic agent [9] and in cold and renal colic treatments [10], while *Leonurus cardiaca* (motherwort) usages include sedative, uterotonic, diuretic, cardiotonic, and hypotensive, as well as bronchial asthma protection [11].

At the scientific level, the bioactive properties of *M. aquatica*, *L. dentata* and *L. cardiaca* have been mostly associated with their phenolic constituents, which are generally major components in extracts obtained with polar solvents (e.g., water, ethanol, methanol, water/alcohol or water/acetone mixtures). The antioxidant capacity of polar extracts from these three plants has been previously tested through chemical antiradical assays [12,13,14,15,16,17,18,19,20]. Besides, neuroprotective effects of methanolic and aqueous extracts of *M. aquatica* aerial parts were shown to counteract oxidative stress in an H_2_O_2_-induced toxicity model in PC12 cells, along with the high ability to inhibit monoamine oxidase (MAO) and a moderate affinity towards GABA_A_ receptor [6,21]. Besides, hydroethanolic extracts of this plant were reported to exhibit promising antiproliferative and anti-inflammatory activities, as demonstrated in MCF-7 human breast cancer cells [22] and in an in-vivo mice model [7], respectively.

In contrast to *M. aquatica*, *L. dentata* extracts have been scarcely studied. Still, promising antiproliferative and apoptotic activities were demonstrated in MCF-7 cells [18]. Moreover, the anti-inflammatory effects of hydromethanolic extracts from this plant were evidenced using bone marrow-derived macrophages and murine epithelial cell lines, as well as in mice [18]. Notably, the anti-inflammatory activity of *L. cardiaca* was also suggested by Flemmig et al., when demonstrating the high lactoperoxidase activity of 70% ethanol extracts [23]. Besides, high in-vitro immunomodulatory potential was reported for an hydroacetonic extract in HUVECs cells due to their effects on viability, apoptosis, NO^•^ production, cytotoxicity, and platelet-activating factor secretion. This extract has also shown to be capable of reducing platelet aggregation [20].

Taking into account the claimed antioxidant and anti-inflammatory potential of *M. aquatica*, *L. dentata* and *L. cardiaca* polar extracts, we decided to evaluate the hepatoprotective activities of hydroethanolic extracts obtained from these three plants and investigate the relationship between their antioxidant properties and their specific phenolic composition. Interestingly, in contrast to previous studies on these extracts that were mostly obtained from wild plants, the botanical samples herein used have been cultivated under an organic regime, which is currently pointed as one important market strategy [24]. In fact, organic cultivation is recognized as a sustainable agricultural system, and medicinal and aromatic products produced under this system are readily accepted in global markets and command higher prices than those grown with chemical inputs [7], also claiming to contain higher amounts of specific phytochemicals, namely phenolic compounds [25,26].

## 2. Materials and Methods

### 2.1. Chemicals

BHA (butylated hydroxyanisole) and DPPH^•^ (2,2-diphenyl-2-picrylhydrazyl radical) were obtained from Sigma Chemical Co (St Louis, MO, USA). Porcine trypsin was purchased from Roche (Barcelona, Spain). Tripan blue, dimetilsulfoxide (DMSO), “Minimum Essential Medium Eagle (MEM)” and RPMI-1640 culture media, mix of antibiotics and antimycotic, sodium piruvate, sodium bicarbonate, 3-(4,5-dimethylthiazol-2-yl)-2,5-diphenyltetrazolium bromide (MTT), dichlorofluorescein diacetate, cisplatin, and potassium dichromate were purchased from Sigma-Aldrich (Madrid, Spain) and fetal bovine serum (FBS) was obtained from T.D.I. (Madrid, Spain). The phenolic standards eriodictyol-7-*O*-glucoside, naringenin-7-*O*-glucoside, luteolin-7-*O*-glucoside, quercetin-7-*O*-rutinoside, rosmarinic acid, and verbascoside were obtained from Extrasynthese (Genay Cedex, France). Ascorbic acid, formic acid and ethanol were purchased from Panreac (Barcelona). *n*-Hexane, methanol and acetonitrile, all with HPLC purity, were purchased from Lab-Scan (Lisbon, Portugal).

### 2.2. Plant Material and Obtention of Phenolic-Rich Extracts

Aerial parts (leaves and stems) of *M. aquatica* and *L. cardiaca* and flowers of *L. dentata* were purchased from Ervital (Mezio-Castro Daire, Portugal, GPS coordinates 40.976351, −7.903492), a pioneer company in Portugal in organic farming, specialized in the production and commercialization of aromatic and medicinal plants using an organic regime growth in fields of *Serra do Montemuro*, located in *Montemuro* region at about 1000 m of altitude. The plants have been cultivated under the general conditions described by Afonso et al. [27]. After collection, the aerial parts were dried in a ventilated incubator at 20 to 35 °C for 3 to 5 days.

The extracts were prepared after defatting the plant powder with *n*-hexane, (1:30), following the general procedure described by Pereira et al. [28] i.e., the defatted residue was extracted six times with 150 mL of 80% ethanolic solution (*v*/*v*) at room temperature for 1 h.

The resulting dispersions were combined, filtrated through a G3 sintered plate filter, concentrated, and then treated in order to obtain phenolic-rich extracts [28]. In detail, approximately 0.4 g of each ethanolic extract was dissolved in 15 mL of water and eluted in three Strata SPE C18-E cartridges (2 g, Waters, Milford, MA, USA). The cartridges were then washed three times with 30 mL of water, and the phenolic compounds were recovered by elution with 20 mL of methanol. The resulting extracts were concentrated in a rotary evaporator, frozen at −20 °C, freeze-dried, and kept under vacuum in a desiccator in the dark.

### 2.3. Evaluation of Radical Scavenging and Inhibition of 5-Lipoxygenase Activities

#### 2.3.1. Reducing Power, DPPH^•^ Scavenging Activity

The ability of *M. aquatica* (0.025–0.25 mg/mL), *L. dentata* (0.05–0.25 mg/mL) and *L. cardiaca* (0.05–0.25 mg/mL) ethanolic extracts in reducing iron (III) was assessed by the method described by Catarino et al. [29]. In more detail, the different concentrations of each extract were mixed with 2.5 mL of 0.2 M phosphate buffer (pH 6.6) and 2.5 mL of 1% potassium hexacyanoferrate [K_3_Fe(CN)_6_] aqueous solutions. After 20 min of incubation at 50 °C, 2.5 mL of 4% trichloroacetic acid was added followed by vigorous stirring. After that, 2.5 mL of each solution was transferred to new vials where 2.5 mL of deionized water and 0.5 mL of 0.1% of iron chloride (FeCl_3_) were added, and the absorbance was then measured at 700 nm. A linear regression analysis was carried out by plotting the mean absorbance against the concentrations, and the EC_50_ value was determined considering the extract concentration that provides 0.5 of absorbance. BHA was used as a reference compound.

The capacity of *M. aquatica* (0.025–0.25 mg/mL), *L. dentata* (0.05–0.5 mg/mL) and *L. cardiaca* (0.05–0.5 mg/mL) to scavenge DPPH radical was performed according to the procedure previously described [30]. Briefly, 0.1 mL of different concentrations of the extracts was prepared and added to 1.9 mL of a methanolic solution of DPPH^•^, followed by vigorous stirring. After 30 min of incubation in the dark, the absorbance of the mixtures was measured in a spectrophotometer at 517 nm, against a blank (absence of DPPH^•^). The radical scavenging activity of each ethanolic extract was calculated as the percentage of DPPH^•^ discoloration: % DPPH^•^ scavenging = (A_c(0)_ − A_e(t)_)/A_c(0)_ × 100, where: A_c(0)_ = Absorbance of the control at *t* = 0 min; A_e(t)_ = Absorbance of the extract at *t* = 30 min. Based on the graphic values of the percentage of DPPH radical inhibition vs. extract concentration, the EC_50_ (concentration of the extract able to inhibit the 50% of the DPPH) of each extract was calculated. Ascorbic acid was used as a reference.

#### 2.3.2. NO^•^ Scavenging Test

The ability of extracts in scavenging NO^•^ followed the methodology previously reported by Afonso et al. [31]. In brief, 100 μL of sodium nitroprusside (3.33 mM) in PBS 100 mM (pH = 7.4) were added with 100 μL of the different sample concentrations (0.02–1 mg/mL) of each extract and incubated for 10 min at room temperature under light irradiation. Sodium nitroprusside is known to decompose in aqueous solution at physiological pH (7.2), producing NO^•^, and this, in turn, interacts with molecular oxygen, producing NO_2_^−^, which in the presence of Griess reagent (100 µL, 1% of sulfanilamide and 0.1% of naphthylethylenediamine dihydrochloride in 2.5% of phosphoric acid) produces a purple azo dye. The measurement of the absorbance was determined spectrophotometrically at 562 nm. The EC_50_ value for the NO^•^ scavenging activity was determined by plotting the percentage of inhibition of nitrite generation in the presence of the plant extracts against the tested concentrations. Ascorbic acid was used as the reference compound.

#### 2.3.3. Inhibition of 5-Lipoxygenase (5-LOX)

The 5-LOX inhibitory assay was performed in a quartz 96-well plate according to the procedure of Afonso et al. [31], with some modifications. For that, 20 µL of the extract sample solutions (0.1–1.0 mg/mL for *M. aquatica*, 0.2–3.0 mg/mL for *L. dentata* and 0.2–3.0 mg/mL for *L. cardiaca*) and 20 µL of the 5-LOX work solution were added to each well and incubated at 37 °C in the plate reader for 10 min. The reaction was then initiated by the addition of 40 µL of pre-heated linoleic acid (500 µM) and the formation of (*9Z*, *11E*)-(*13S*)-13-hydroperoxyoctadeca-9,11-dienoate was followed for 20 min taking measurements every minute at 234 nm. The reaction rate at each inhibitor concentration was calculated by determining the slope of the experimental values and the percentage of inhibition by the following formula.

$${\%}_{inhibition}=\frac{v_{0}-v_{\left[inhibitor\right]}}{v_{0}}\times 100$$

where $v_{0}$ corresponds to the reaction rate of control and $v_{\left[\mathrm{inhibitor}\right]}$ to the reaction rate of the extract. EC_50_ corresponds to the concentration of the tested extract able to inhibit the hydrolysis of the substrate (linoleic acid) by about 50% (EC_50_). Ascorbic acid was used as the reference.

### 2.4. Evaluation of Biological Activity in Cellular Assays

#### 2.4.1. Cell Culture and Treatments

The general cell culture procedures and those of MTT and ROS experiments followed the procedures previously described by Pereira et al. [30]. The hydroethanolic extracts of *M. aquatica*, *L. dentata* or *L. cardiaca* (final concentrations of 1–200 μg/mL) and/or potassium dichromate (K_2_Cr_2_O_7_) (final concentrations of 1.5, 5, 25 or 500 μM) were dissolved in the culture medium. Twenty-four hours after seeding, the cells’ culture medium was replaced by fresh culture medium containing the treatment agents and the cells were maintained in culture, for 6 h or 72 h (in the MTT assay, to measure acute and long-term toxicity, respectively) or 48 h (intracellular ROS).

#### 2.4.2. MTT Assay

The viability of HepG2 cells was estimated by the formazan formation from the tetrazolium salt by living cells, as previously described [30,32]. Cell viability was calculated as the percentage of living cells compared to untreated (control) cells. Cisplatin (0.3 to 30 μg/mL) was used as a positive control.

#### 2.4.3. Intracellular ROS production

ROS production was analyzed by flow cytometry following the procedure described by Pereira et al. [30]. After 48 h incubation of HepG2 cells with the hydroethanolic extracts and/or potassium dichromate, the medium was replaced by RPMI culture medium containing 5 μg/mL of the probe 2,7-dichlorofluorescein diacetate (DCFH-DA) for 30 min, and then cells were trypsinized and resuspended in FBS free-medium. ROS generation was measured and analyzed in a cytometer FACSort flow cytometer (BD Biosciences, San Jose, CA, USA) with CellQuest software (BD Biosciences). The values were normalized to the percentage of ROS formation by the untreated cells.

### 2.5. Analysis of the Phenolic Compounds

The analysis of phenolic compounds was performed by liquid chromatography under the general conditions previously described by Catarino et al. [29]. Gradient elution was carried out with a mixture of 0.1% (*v/v*) of formic acid in water (solvent A) and acetonitrile (solvent B), starting from 10 to 20% of solvent B over 6 min, from 20 to 25% of solvent B over 12 min, from 25 to 34% over 30 min, increasing to 100% at 37 min maintaining for 3 min, followed by the return to the initial conditions at 40 min. The identification of phenolic compounds was corroborated by MS analysis using a Thermo LTQ XL (Thermo Scientific, USA) ion trap MS apparatus equipped with an ESI source, operating in negative mode, under the same conditions as previously described [28]. Additionally, the quantification of the majority of the compounds in three plant extracts was performed by peak integration using the external standard method, with the exact or structurally-related standard compounds. Considering the nature of the phenolic compounds, eriodictyol-7-*O*-glucoside was used to quantify eriodictyol-*O*-rutinoside and hesperetin-7-*O*-rutinoside; naringenin-7-*O*-glucoside was used to quantify naringenin-7-*O*-rutinoside; luteolin-7-*O*-glucoside was used to quantify luteolin glycosides (peaks 6, 10 and 12); Flavonols (peaks 3, 9 and 11) were quantified with quercetin-7-*O*-rutinoside; Caffeic acid derivatives (peaks 1 and 16) were quantified with rosmarinic acid; while verbascoside was used to quantified the phenylethanoid glycosides (peaks 5, 7, 14 and 15). The linearity of the calibration curves, the regression coefficient (R^2^) and the detection and quantification limits (LOD and LOQ, respectively) are represented in Table S1.

### 2.6. Statistical Analysis

All statistical analyses were performed with GraphPad Prism 6 statistical software (GraphPad, San Diego, CA, USA). Data were expressed as mean ± S.D. or as mean ± S.E.M. of three to four independent experiments performed at least in triplicate. The comparison between groups was performed by one-way ANOVA, followed by Tukey’s test. Unpaired Student’s *t*-test was used for comparison between two groups. Correlation analyses were performed using a two-tailed Pearson’s correlation test. A difference was considered statistically significant when *p* < 0.05. For cellular assays, * *p* < 0.05; *** *p* < 0.001 when compared to cells exposed to potassium dichromate in the absence of extracts; ### *p* < 0.01; #### *p* < 0.0001 when compared to untreated cells.

## 3. Results and Discussion

### 3.1. Antioxidant and Anti-Inflammatory Properties (in Chemical Models)

The hydroethanolic extracts of *M. aquatica*, *L. dentata* and *L. cardiaca* exhibited promising antioxidant potential, as evaluated by DPPH^•^ and reducing power assays, with potencies order of *M. aquatica* > *L. dentata* > *L. cardiaca* and EC_50_ values ranging from 8.1 ± 1.3 to 18.3 ± 1.5 (corresponding to about three to seven times higher than that of ascorbic acid) and from 51.9 ± 12.6 to 94.7 ± 12.1 (about two to three times higher than that of BHA, used as the standard) (Table 1), respectively. In general, the herein registered EC_50_ values for DPPH^•^ are promising when compared to previous literature data for methanol, hydromethanolic or aqueous (at 50 °C) extracts of *M. aquatica* (27–50.0 μg/mL corresponding to 4–15 times that of ascorbic acid) [20,32], of *L. dentata* (48.7–71.1 μg/mL equivalent to 24-fold that of ascorbic acid) [17,18] and *L. cardiaca* (12–144 μg/mL) [33,34].

The ability of *M. aquatica*, *L. dentata* and *L. cardiaca* hydroethanolic extracts to counteract inflammatory events was evaluated by NO^•^ scavenging and 5-LOX inhibitory methods. NO^•^ is a chemical mediator generated in biological tissues, while 5-LOX acts as a key enzyme in leukotrienes biosynthesis. These molecules are potent mediators involved in various physiological processes and are related to inflammatory conditions. As shown in Table 1, relevant activity was only observed for the *M. aquatica* extract, particularly regarding its ability to scavenge NO^•^, which corresponded to about a quarter of that of ascorbic acid (EC_50_ of 217.0 ± 19.0 and 92.0 ± 7.3 μg/mL, respectively). Please note that the in-vivo anti-inflammatory effect has been previously described for *M. aquatica* [7] and *L. dentata* hydroalcoholic extracts [18], although authors did not elucidate the molecular mechanisms behind their effects. Hence, our results seem to suggest that, while NO^•^ scavenging and LOX inhibition might be partially involved in the inflammatory protection of *M. aquatica* extract, they do not play a role in the case of *L. dentata* and *L. cardiaca* extracts.

### 3.2. Hepatoprotective Activities in HepG2 Cells

**Figure 1:**
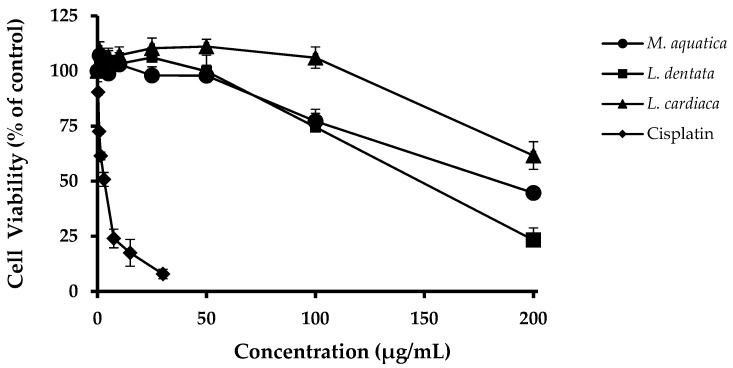
Viability of HepG2 cells exposed for 72 h to a concentration range of cisplatin (0.3 to 30 µg/mL), used here as a toxic positive control, or of hydroethanolic extracts (1 to 200 µg/mL) obtained from *M. aquatica*, *L. dentata* and *L. cardiaca*. Values are means ± SD of the percentage of cell viability with respect to control untreated cells.

The human hepatocarcinoma cell line HepG2, which retains many specialized functions of normal human hepatocytes, was used because this is a common model to screen possible protective effects of compounds/extracts in liver cells. The MTT assay performed after 72 h exposure of these cells to *M. aquatica, L. dentata* and *L. cardiaca* hydroethanolic extracts (1–200 μg/mL) allowed to conclude that the latter was the one that least affected their metabolic activity (Figure 1). In fact, while this extract only caused a negative impact on the viability of HepG2 cells at concentrations >100 μg/mL, the toxic limit for *M. aquatica* and *L. dentata* was lower (50 μg/mL).

Considering the above results, the comparison of the potency of the three plant extracts in protecting HepG2 from ROS generation and cell death was further investigated at 50 μg/mL [29]. In this and previous studies, we have used potassium dichromate (K_2_Cr_2_O_7_) as a model aggressive agent because of its ability to generate oxidative stress and interact with intracellular macromolecules causing DNA damage [35]. K_2_Cr_2_O_7_ affects HepG2 cells through multiple mechanisms, which overall cause a massive increase of ROS and ultimately lead to cell death through apoptosis and necrosis. These effects are dependent on the time and levels of exposure to the toxic substance [5,30,36,37].

**Figure 2:**
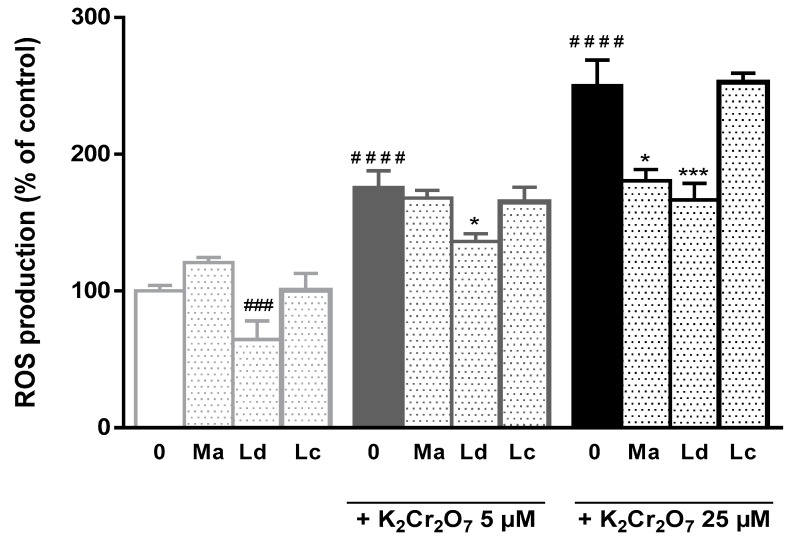
Effect of *M. aquatica*, *L. dentata* and *L. cardiaca* hydroethanolic extracts (at 50 µg/mL) in ROS production in HepG2 cells in the absence or presence of 5 or 25 μM of potassium dichromate. Values are expressed as means ± S.D. of percentage of ROS production with respect to the control (untreated cells). K_2_Cr_2_O_7_, potassium dichromate; Ma, *M. aquatica* extract; Ld, *L. dentata* extract; Lc, *L. cardiaca* extract.

As shown in Figure 2, the treatment of HepG2 with K_2_Cr_2_O_7_ at 5 μM and 25 μM deregulated ROS production *versus* cellular antioxidant balance, resulting in increased ROS intracellular levels by over 1.7-fold and 2.4-fold, respectively. These effects could be counteracted by the presence of the plant extracts, particularly from *M. aquatica* and *L. dentata* origin. Of note, regardless, being less active than *M. aquatica* extract in DPPH^•^ and reducing power assays, *L. dentata* extract showed the highest effectiveness in attenuating ROS formation in HepG2 cells. Thus, a significant decrease in the rate of K_2_Cr_2_O_7_ induced ROS production (23% and 31%, at 5 μM and 25 μM, respectively), as well as under non-stimulated conditions of ROS production (36%) was observed. In contrast, the protective effects of *M. aquatica* were only significant for the most-toxic conditions, i.e., during incubation with 25 μM K_2_Cr_2_O_7_.

**Figure 3:**
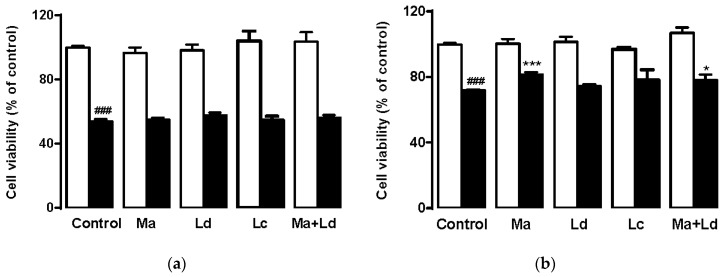
Effect of *M. aquatica*, *L. dentata* and *L. cardiaca* hydroethanolic extracts on HepG2 viability incubated with 500 μM or 1.5 μM K_2_Cr_2_O_7_ for 6 h (**a**) or 72 h (**b**), respectively, in absence (white bars) or in presence (black bars) of K_2_Cr_2_O_7_. Values are expressed as means ± S.D. of percentage of cell viability with respect to control (untreated cells). Ma, *M. aquatica* extract; Ld, *L. dentata* extract; Lc, *L. cardiaca* extract.

The possible protection activity of extracts towards loss of cell viability induced by K_2_Cr_2_O_7_ was also tested in two cytotoxic models, namely 500 μM K_2_Cr_2_O_7_ for 6 h (acute toxicity) and 1.5 μM K_2_Cr_2_O_7_ for 72 h (long-term toxicity) [38]. Overall, the exposure to the toxic agent caused a decrease in cell viability of about 45% and 30%, respectively (Figure 3). These results showed that none of the extracts could prevent the loss of viability in cells cultured under a strong toxic treatment but still, both *M. aquatica* extract (50 μg/mL) and *M. aquatica*/*L. dentata* mixture (25 μg/mL each) were able to protect the cells from a mild toxic insult. Our results suggest that *M. aquatica* does effectively reduce the levels of ROS in liver cells. No clear inhibition of ROS production was observed when this was induced by low concentrations of K_2_Cr_2_O_7_, nevertheless, at higher concentrations and hence higher levels of oxidative stress, a significant antioxidant effect of *M. aquatica* extract was seen (Figure 2). Since *M. aquatica* and *L. dentata* were able to reduce ROS production induced by K_2_Cr_2_O_7_, but only *M. aquatica* partially prevented cell death induced by a mild dose of the toxic compound over the long term, this suggests that the cytoprotective effect of *M. aquatica* could be mediated through ROS-dependent scavenging action; however, this must be confirmed by other assays which evidence the mechanisms. In contrast, the antioxidant protection conferred by *L. dentata* was not translated to an evident defense against the K_2_Cr_2_O_7_-induced long-term toxicity effect.

### 3.3. Phenolic Characterization of M. aquatica, L. dentata and L. cardiaca Hydroethanolic Extracts

Considering that the bioactive properties of *M. aquatica*, *L. dentata* and *L. cardiaca* are mainly claimed to be associated with their phenolic constituents [7,18,34], the phenolic composition of the three extracts was herein evaluated. Overall, the total amounts of phenolic compounds in the extracts followed the sequence order of *L. cardiaca* > *M. aquatica* > *L. dentata*, with values of 500 ± 49, 307 ± 29 and 94 ± 4 μg/mg, respectively (data not shown). Moreover, their phenolic profiles were quite different to each other (Figure S1 and Table 2).

Thus, while the extract from *M. aquatica* origin was mainly composed of flavanones, such as eriodictyol, naringenin and hesperitin glycosides, *L. dentata* extract was rich in rosmarinic acid, reaching up to 72% of the total quantified phenolic compounds. In turn, phenylethanoid glycosides comprised the most prevalent phenolics in *L. cardiaca* extracts, specially lavandulifolioside and verbascoside (254 ± 36 and 137 ± 20 μg/mg extract, respectively). In general, the chemical composition of the extract analyzed in the present study agreed with previous reported data regarding these three plants [18,23,39,40]. Nevertheless, this is the first time that leucoseptoside A (MW 638, 31.5 ± 4.6 μg/mg extract) and leonoside B (MW 784, 25.1 ± 4.7 μg/mg extract) have been detected in *L. cardiaca* extracts.

Moreover, it must be highlighted that regardless of being the richest in total phenolic compounds, the *L. cardiaca* extract showed weak biological activity, both in the chemical and cellular models used here, thus indicating that their main phenolic compounds have low antioxidant and cytoprotective properties. In turn, the main phenolic components of *M. aquatica* or *L. dentata* extracts can in part be associated with the observed hepatoprotection due to antioxidant and additional mechanisms. Table 3 summarizes the Pearson correlation coefficients between the amounts of classes of phenolic components or major individual phenolic compounds found in the three hydroethanolic extracts and the results obtained in radical scavenging, 5-LOX inhibition assays and biological experiments.

The most abundant hydroxycinnamic acid present in *L. dentata* and *M. aquatica* extracts, i.e., rosmarinic acid, was well correlated with the antioxidant effect measured by DPPH^•^ (0.815) and ROS-scavenging protection in HepG2 cells (0.996). Indeed, rosmarinic acid has demonstrated to possess high scavenging ability via direct and indirect ROS-scavenging activities, including in the cell model herein used [30,41,42]. Additionally, rosmarinic acid is a strong scavenger of ONOO^−^ and other free radicals [3,43].

Concerning flavonoid compounds, the antioxidant effects measured by reducing power and for counteracting DPPH^•^ and NO^•^ showed to be in good agreement with the content of flavones and flavanones, with Pearson correlation coefficients of about 0.9. Of note, *L. dentata* and *M. aquatica* extracts contained moderate amounts of luteolin derivatives, and overall, the content of these flavones has a good correlation with almost all biological assays carried out in the present study. These data are consistent with the available literature, in which luteolin has been described as a cytoprotective agent in HepG2 and PC12 cells, in part due to its ROS scavenging activity [30,44].

The cytoprotective effect found in our assays was reasonably well correlated with the amounts of flavanones (0.803), which could explain the high activity of *M. aquatica* hydroethanolic extract, mainly composed of eriodictyol-7-*O*-rutinoside. In fact, it has been described that this compound has a lipoxygenase inhibitor action [45], and eriodictyol has been reported to reduce NO^•^ production in macrophages [27,46]. Previous studies have also shown the efficacy of eriodictyol in ROS scavenging activity in HepG2 [30] and retinal cells [47]. In addition, a cytoprotective effect under similar conditions to these used in the present work has been reported [30]. Finally, *M. aquatica* hydroethanolic extract could exert effective protection through interference with proapoptotic events that lead to cell death, because eriodictyol is a potent inhibitor of key apoptotic steps, such as pro-caspase-3 and pro-caspase-9 cleavage and the release of cytochrome C [46,48].

## 4. Conclusions

The present study suggests the potential beneficial effect on liver cells of hydroethanolic extracts obtained from *M. aquatica* and *L. dentata* cultivated under an organic cultivation system. While *M. aquatica* extract exerts a cytoprotective effect on HepG2 cells, *L. dentata* extract has ROS-scavenging efficiency. Focusing on the phenolic profiles of the most-active extracts, their content in flavanones and in rosmarinic acid is possibly associated with the antioxidant and hepatoprotective effects of *M. aquatica* and *L. dentata*, respectively, and the extracts are proposed as valuable sources of natural metabolites with potential health-benefit properties.

## Figures and Tables

**Table 1:** Yield of extraction (%), antioxidant and anti-inflammatory activities (EC_50_, µg/mL) of *M. aquatica, L. dentata* and *L. cardiaca* extracts.

Sample	Yield (%)	DPPH Scavenging (μg/mL)	Reducing Power (μg/mL)	NO^•^ Scavenging (μg/mL)	5-LOX Inhibition (μg/mL)
*M. aquatica*	11.3	8.1 ± 1.3 ^a^	51.9 ± 12.6 ^a^	217.0 ± 19.0 ^a^	174.5 ± 30.5 ^a^
*L. dentata*	3.2	11.6 ± 1.1 ^b^	78.9 ± 2.6 ^b^	879.3 ± 192.8 ^b^	237.9 ± 15.2 ^b^
*L. cardiaca*	3.7	18.3 ± 1.5 ^c^	94.7 ± 12.1 ^b^	>1000	>1000
AA	-	2.5 ± 0.4 ^d^	-	92.0 ± 7.3 ^c^	7.8 ± 1.0 ^c^
BHA	-	-	27.1 ± 1.2 ^c^	-	-

Mean values ± SD. Statistical analysis was performed by one-way ANOVA, followed by a Tukey’s test. In each column, different letters (a–d) mean significant differences (*p* < 0.05); RP: Reducing Power; AA: Ascorbic acid; BHA: Butylated hydroxyanisole; 5-LOX: 5-lipoxygenase.

**Table 2:** Phenolic compounds of *M. aquatica, L. dentata* and *L. cardiaca* hydroethanolic extracts determined by UHPLC-DAD-ESI-MS^n^.

-					Plant Extract		
					*M. aquatica*	*L. dentata*	*L. cardiaca*
Peak	RT (min)	λmax (nm)	Compound	ESI—MS^n^ Fragmentation (*m*/*z*)	Phenolic Content (μg/mg of extract)		
	*Caffeic acid derivatives*						
1	8.2	290,329	Caffeic acid glc	341→179→135	-	-	3.7 ± 0.8
16	21.1	290,328	Rosmarinic acid	359→179→135, 161	64.2 ± 8.8	67.8 ± 6.7	-
	*Phenylethanoid glycosides*						
5	16.7	290,329	Lavandulifolioside	755→593→461→315	-	-	253.6 ± 35.8
7	17.5	290,329	Verbascoside	623→461→315→135	-	-	137.4 ± 19.9
14	19.4	ND	Leucoseptoside A	637→461→315→135	-	-	31.5 ± 4.6
15	20.0	ND	Leonoside B	783→607→475→329	-	-	25.1±4.7
	*Flavones*						
6	16.8	254,267,345	Luteolin-7-*O*-rut	593→285→241	43.3 ± 10.0	-	-
10	18.1	253,267,345	Luteolin-7-*O*-glcA	461→285→241	D	26.2 ± 4.0	-
12	18.9	ND	Luteolin-7-*O*-rut	593→285→257	-	-	D
17	21.1	266,329	Apigenin-7-*O*-gl	431→269→225	-	D	-
18	26.3	266,330	Apigenin-7-*O*-(6″ acetyl)glc	473→269, 413	-	D	-
	*Flavonols*						
2	8.2	ND	Rutin-*O*-glc	771→609→301	-	D	-
3	13.7	ND	Quercetin-3-*O*-soph	625→301→179	-	-	5.7 ± 1.1
9	18.0	256,267,355	Rutin	609→301→179	-	-	15.8 ± 2.1
11	18.9	256,267,357	Quercetin-3-*O*-glc	463→301→179	-	-	24.9 ± 3.8
	*Flavanones*						
4	15.0	283,325sh	Eriodictyol-*O*-rut	595→287→151→107	144.6 ± 22.4	-	-
8	17.9	282,333sh	Naringenin-7-*O*-rut	579→271→151	24.4 ± 3.7	-	-
13	19.5	283,325sh	Hesperetin-7-*O*-rut	609→301→286→241	25.9 ± 3.6	-	-

D, Detected; Glc, Glucoside; GlcA, Glucuronide; RT, retention time; Rut, Rutinoside; Soph, Sophoroside; Mean values ± standard deviations; Numbers correspond to the UHPLC-DAD-ESI-MS^n^ peaks described in chromatograms in Figure 1. Supplementary Material S1.

**Table 3:** Correlation coefficients between the amounts of phenolic components of *M. aquatica, L. dentata* and *L. cardiaca* hydroethanolic extracts and the data from the radical scavenging, 5-LOX inhibition assays and biological experiments.

Assay	Flavan	Flav	Flavo	PEG	RAc
DPPH	0.889	0.986	−0.841	−0.841	0.815
RP	0.973	0.917	−0.688	−0.688	0.653
NO	0.990	0.878	−0.620	−0.620	0.582
ROS	0.373	0.856	−0.990	−0.990	0.996
CytP	0.803	0.284	0.115	0.115	−0.161

Values expressed as Pearson Correlation Coefficient *R*; CytP—Cytoprotection (MTT 72h); PEG—Phenylethanoid glycosides; DPPH—DPPH radical scavenging activity; Flav—Flavones; Flavan—Flavanones; Flavo—Flavonols; NO—Nitric oxide radical scavenging capacity; RP—reducing power potential; RAc—Rosmarinic Acid; ROS—Protection form ROS production (25 μM K_2_Cr_2_O_7_).

## Supplementary Materials

The following are available online at https://www.mdpi.com/2076-3921/8/8/267/s1, Figure S1: Chromatographic representation of *Mentha aquatica* (a), *Lavandula dentata* (b) (both at 280 nm) and *Leonurus cardiaca* (c) (at 340 nm) hydroethanolic extracts, Table S1: Linearity, LOD and LOQ of standard compounds used as references.
