# Supplementary material for: Hepatoprotection of Mentha aquatica L., Lavandula dentata L. and Leonurus cardiaca L
Source: Antioxidants (Basel). 2019 Aug 2;8(8):267. doi: 10.3390/antiox8080267 (PMC6719046; doi:10.3390/antiox8080267)
Supplement: Supplementary file 1 [file antioxidants-08-00267-s001.pdf]

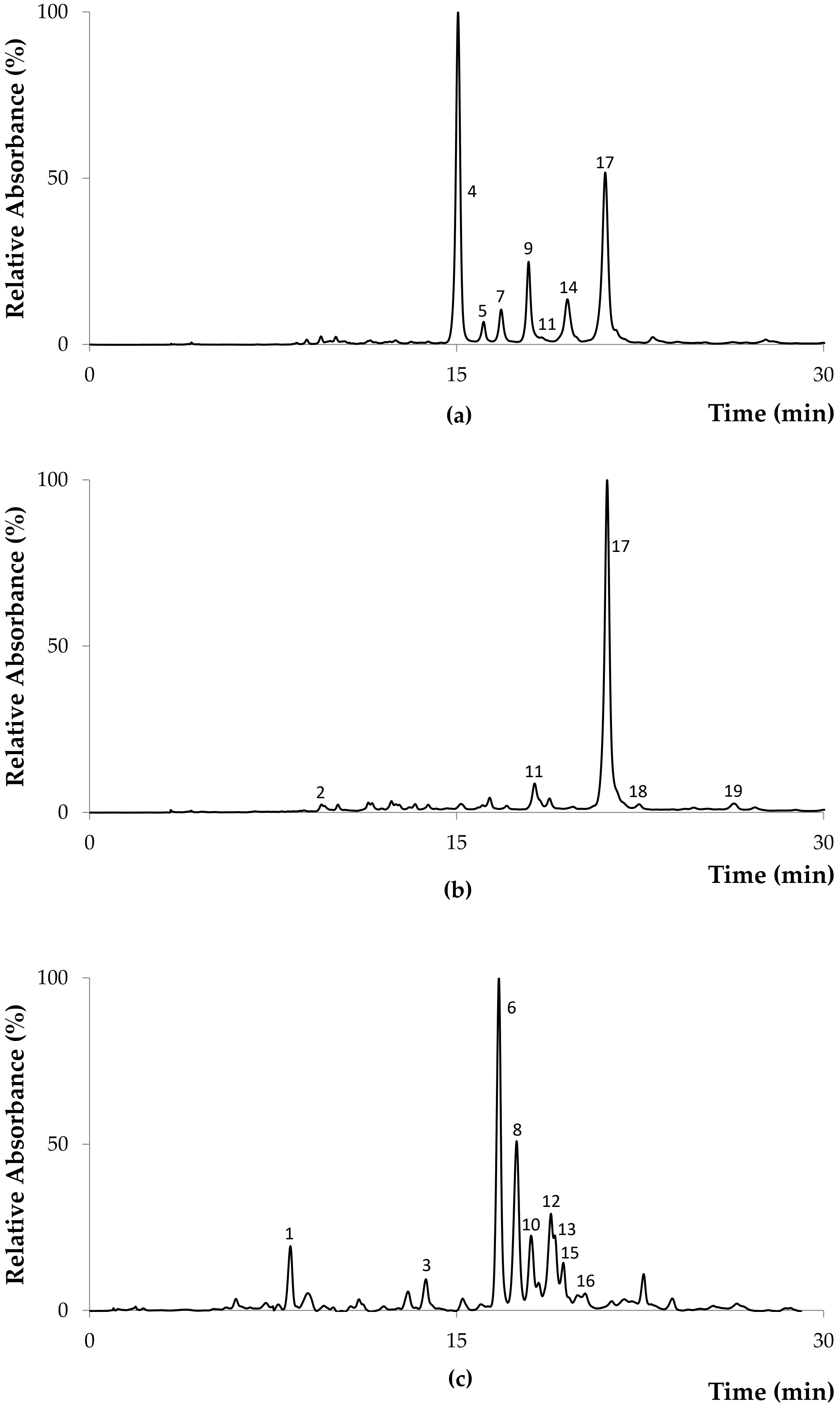

Relative Absorbance (%)
100
50
0
0
15
30
4
5
7
9
11
14
17
(a)
Time (min)
Relative Absorbance (%)
100
50
0
0
15
30
2
11
17
18
19
(b)
Time (min)
Relative Absorbance (%)
100
50
0
0
15
30
1
3
6
8
10
12
13
15
16
(c)
Time (min)

Figure 1. Chromatographic representation of *Mentha aquatica* (a), Lavandula dentata (b) (both at 280 nm) and *Leonurus cardiaca* (c) (at 340 nm) ethanolic extracts. Numbers in the figure correspond to the UHPLC-DAD-ESI-MS^n peaks described in Table 2.

Table 1. Linearity, LOD and LOQ of standard compounds used as references.

Standard Compound	Range concentration (µg/mL)	n[a]	Slope [b] (area counts/mg)	Intercept [b] (area counts /mg)	R^2	LOD (µg/mL)	LOQ (µg/mL)
E-7O-G	10 - 136	5	1106(±10) x 10^4	34(±8) x 10^3	0.9995	5.6	16.9
N-7O-G	5 - 68	5	136(±1) x 10^5	2(±4) x 10^3	0.9991	2.7	8.1
L-7O-G	45 - 473	5	385(±10) x 10^4	7(±2) x 10^4	0.9945	40.6	123.2
RA	15 - 173	5	143(±1) x 10^5	-10(±1) x 10^4	0.9992	6.4	19.3
Verb	44 - 700	5	166(±2) x 10^4	6(±7) x 10^3	0.9985	31.9	96.7
Q-7O-R	12-200	5	48(±1) x 10^5	-3(±8) x 10^3	0.9981	14.4	43.6

E-7O-G, eriodictyol-7-*O*-glucoside; N-7O-G, naringenin-7-*O*-glucoside; L-7O-G, luteolin-7-*O*-glucoside; RA, rosmarinic acid; Verb, verbascoside; Q-7O-R, quercetin -7-*O*-rutinoside

[a] Number of points used for the regression of standard solutions. Injections were done in triplicate.

[b] The standard deviation in the slope and intercept of the regression line is shown in parenthesis
